# Applications of Nondominated Sorting Genetic Algorithm II Combined with WKNN Online Matching Algorithm in Building Indoor Optimization Design

**DOI:** 10.1155/2022/7509659

**Published:** 2022-02-16

**Authors:** Xiwen Yu, Shaoxuan Wang, Feng Xiao

**Affiliations:** ^1^School of Arts and Media, Hefei Normal University, Hefei, Anhui 230601, China; ^2^Shanghai Eigencomm Technologies Ltd., 298 Xiangke Road, 7/F, 707, Pudong New District, Shanghai 201210, China; ^3^Faculty of Information and Engineering, Anhui Vocational and Technical College, Hefei, Anhui 230031, China

## Abstract

The present work aims to improve the comfort of architectural interior design and reduce indoor energy consumption. The Weight K-Nearest Neighborhood (WKNN) algorithm and Nondominated Sorting Genetic algorithm are proposed to locate and analyze the spatial location of indoor personnel and optimize the indoor energy consumption in combination with residential behavior. Firstly, the indoor human behavior data and energy-saving problems are analyzed based on residential behavior theory and architectural physics. The indoor positioning algorithm is proposed to identify the personnel activities to realize the optimization of indoor energy distribution. Secondly, mean filtering and cluster analysis are adopted to optimize sampling points' data and fingerprint database to eliminate data noise. Besides, the WKNN algorithm is used for Wireless Fidelity (Wi-Fi) indoor location fingerprint location. Then, aiming at the multiobjective optimization problem of building indoor energy consumption, the Nondominated Sorting Genetic algorithm obtains the optimal solution of the model. Combined with the indoor location information of personnel, the indoor heating and cooling system is monitored and distributed to reduce the energy consumption in the building while ensuring the living comfort of personnel. The test and simulation results demonstrate that the mean filtering algorithm can solve the room's fluctuation problem of Wi-Fi signals. The cluster analysis algorithm can eliminate the data noise of the fingerprint database and improve the positioning accuracy of the positioning algorithm. Moreover, the location result is independent of the number of nodes; the number of sampling points does not affect the location result. The positioning accuracy of the WKNN algorithm is 2 m, and the positioning error rate within 2 m is about 60%. Compared with other algorithms, the WKNN algorithm has better positioning accuracy and positioning stability. Therefore, the location algorithm designed here can be applied to indoor location optimization. This study provides a reference for optimizing buildings' indoor positioning and energy consumption.

## 1. Introduction

With the improvement of the international economic level, consumers' requirements for the interior design of buildings are constantly improving. Function and space are essential issues in architectural design. Analyzing the coupling relationship between people and architectural space needs to consider people's life behavior and life scene. Residential behavior affects the interior design of buildings and the energy consumption of structures [1, 2]. Parametric design is a new development stage of digital design, which aims to adapt the prototype to the design goal through the collection operation of the prototype. Adjusting the architectural prototype's generation rules and setting variables in the operation process can form the final design result. Especially with the rapid development of information technology, mobile phones, computers, and other information equipment are gradually changing people's living style and potential energy behavior mode, putting forward new requirements for architectural interior design [3].

The booming computer technology promotes the progress of science and technology and provides a more favorable scheme for architectural interior design. The research on detection and information positioning technology, especially for developing spatial positioning technology, mobile sensors, and wireless communication technology, provides adequate technical support for collecting residential behavior information in buildings. The large-scale collection of regular residential behavior information of residents in buildings can analyze the design innovation of residential buildings and the utilization of space in buildings in more detail [4]. On this basis, the energy consumption in the building can also be optimized to ensure the comfort of the indoor environment and reduce the energy consumption of the indoor environment [5].

Here, the fingerprint location method collects and analyzes people's living behavior in the building based on Wireless Fidelity (Wi-Fi) to understand the activity status of residents in the building. Besides, the fingerprint database and sampling data are optimized to improve the location accuracy of the location algorithm. Then, the Nondominated Sorting Genetic algorithm is used to analyze the physical characteristics of buildings to find the optimal solution set of energy consumption allocation optimization to solve the problem of indoor energy consumption. Finally, simulation experiments are carried out to verify the effectiveness of the design algorithm in dealing with the related issues. The innovation of this paper is to consider the activity location of indoor personnel, analyze the impact of personnel on building indoor energy consumption, and reduce building energy consumption by adjusting the distribution stage of the air conditioning system. Furthermore, using computer technologies reduces building energy consumption while considering residential behavior, which achieves satisfying results.

This paper primarily consists of four sections.  Section 1 analyzes the problems of indoor architecture based on residential behavior and puts forward the existing issues in the emergence stage.  Section 2 solves the current problems using a computer algorithm and optimizes the algorithm using mean filtering and clustering algorithms.  Section 3 solves the problem of indoor energy consumption using a multiobjective optimization algorithm.  Section 4 designs experiments to analyze the ability of the algorithm to deal with the related problems and verify the algorithm's effectiveness.

## 2. Literature Review

A considerable number of domestic and foreign scholars have engaged in indoor positioning and building energy consumption optimization. Mukkavaara and Sandberg studied the application of generative design to the architectural design environment. They suggested that this technology allowed designers to use computers to improve architectural design schemes during design; in addition, compared with the genetic algorithm and energy performance model, this technology could effectively explore scheme space problems and provide a general reference framework for architectural design exploration [6]. Borhani Afuosi and Zoghi investigated providing special services for residents of intelligent buildings to improve energy efficiency. The authors employed a low-cost indoor positioning system based on Wi-Fi fingerprints embedded in smartphones to understand the identity information, residence, and the current location of personnel in the building. They also established a radio map information platform and optimized it based on online layer clustering and the K-Nearest Neighborhood (KNN) method. A simulation experiment found that this method increased the error under 2 m by 40% compared to other methods [7]. Guo et al. researched battery parameter identification and optimization technology using the Elman neural network. They established the optimization model by combining the teaching optimization algorithm and differential evolution algorithm and applied it to the parameter optimization process of the battery model. The experimental results showed that the neural network model based on the optimization algorithm could solve the annual salary parameter identification and optimization problems and simulate and predict the equipment under different operating conditions [8]. McLane and Pable explored the impact of the environment involved in transitional housing on people's spiritual recovery by considering residents' age, race, health needs, etc. They also designed and improved the internal space of the building based on social science and spatial analysis theory to reduce residents' sense of marginalization and establish a spatial structure for residents to gather [9].

From the existing research literature, the research on the optimization of architectural interior space design principally focuses on the optimization of architecture, while the research on human-based living behavior is still in the development stage. Therefore, this paper establishes an indoor optimization design scheme suitable for living behavior. A computer algorithm is applied to optimize the indoor design to establish a more humanized living environment and optimize the indoor energy consumption of the building to achieve the purpose of energy-saving.

## 3. Building Indoor Locating and Energy Consumption

### 3.1. Building Indoor Design and Energy Consumption Based on Residential Behaviour

The advancement of urbanization has caused people to spend more time indoors in the building, and the requirements for the comfort and air quality of the indoor environment are also increasing [10]. In a large building, the central air-conditioning system is a vital means to achieve good indoor air circulation. While creating a comfortable environment indoors, it also causes huge energy consumption problems [11]. Therefore, it is very important to reduce the building energy consumption while maintaining a suitable indoor environment [12, 13]. Restrictions on economic development and social progress by the energy crisis could be alleviated by reducing building energy consumption [14]. While the level of society enhances continuously, building energy consumption is also increasing. The accurate calculation of building energy consumption has become a vital factor in building design and exerts an important impact on the development of the entire building industry [15].

Physical architectural performance is the characteristics of buildings on basic physical attributes such as sound, light, and heat. Specifically, it is a specific operating condition of buildings under these attributes. Due to the complexity of the building system, physical architectural performance carries various impacts of buildings on human living behavior and life behavior [16]. Combining the sample data of behavior style with the facility layout, environmental data, and energy consumption data of fixed furniture in the space can obtain the continuous behavior information of people's living behavior in daily life. In this way, the behavior content and frequency of people in the building can be restored to deeply understand the living needs of residents and the function of building space [17].

**Figure 1:**
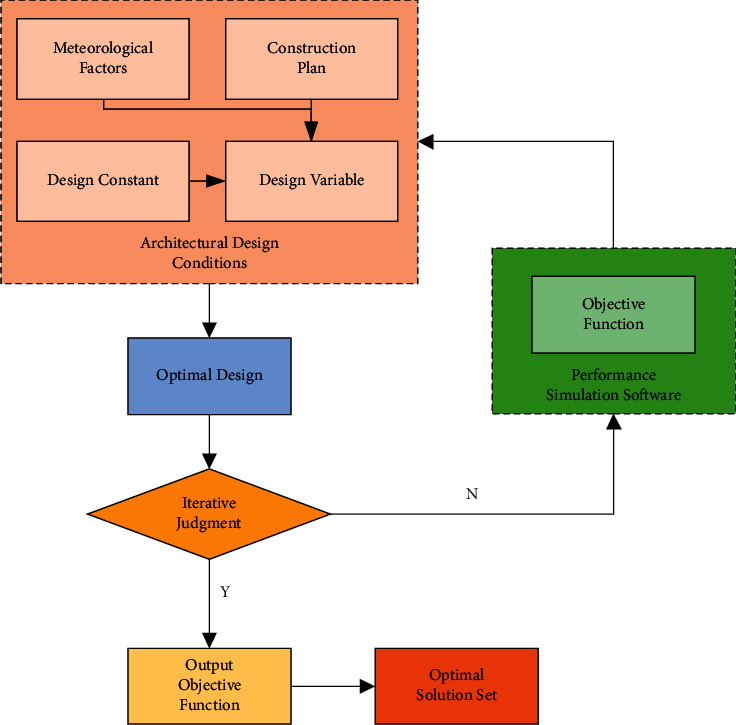
Optimization-driven design of building physical performance.

Residential behavior is the external expression of psychological activities in family life and the transition of living space state. Its external performance results from the interaction between humans and the indoor environment, expressed by the environmental, psychological, and physical, and ecological parameters. The state transition is the spatial expression of life behavior, which graphics can present. The traditional research on building indoor comfort and energy consumption takes a specific variable as the research object for modeling and analysis, so the optimization effect is single and cannot effectively analyze the coupling relationship between them. The architectural space is analyzed based on residential behavior in the research process.  Figure 1 reveals the architectural design process under optimizing building physical performance. As shown in Figure 1, the objective function is established by comprehensively considering various influencing factors. The situation after the optimal design can also be judged again by judging the number of iterations until the output objective function obtains the optimal solution.

During indoor locating, the locating accuracy and the cost and power consumption of locating in the city must be considered. Based on the careful consideration of these issues, technologies that can affect the indoor environment of the building can be analyzed and obtained. In this scenario, the optimization algorithm is to absorb indoor locating and navigation [18]. The Received Signal Strength Indicator (RSSI) based on Wireless Local Area Network (WLAN) locating technology has low locating cost, low energy consumption, and high accuracy, which can access Wi-Fi to apply the technology to indoor environments and perform high-precision locating by judging the signal strength. The indoor locating algorithm based on Wi-Fi technology has the following characteristics: there are enough Wi-Fi access points in the building indoor environment to monitor the signal strength of the mobile device of a specific user [19]. The Wi-Fi signal has the characteristics of long-distance transmission, which can greatly increase the range of locating. In the actual application process, the highest accuracy of Wi-Fi locating can reach 1∼5 m, which can meet the actual needs of users at this stage. Hence, it is the most promising locating technology for large-scale popularization and use.

### 3.2. Wi-Fi Location Fingerprint Locating Algorithm Based on WKNN

The location fingerprint locating algorithm based on Wi-Fi is characterized by low cost, easy implementation, and good location effect, so it has gradually become the mainstream technology of indoor location research. The Wi-Fi fingerprint locating algorithm uses the signal strength in the stored fingerprint database to match the signal strength of the currently connected beaker for location. When collecting network signals, the collected signal needs to be denoised to reduce the impact of signal noise on positioning accuracy [20]. However, most positioning algorithms do not consider noise reduction of collected signals and rarely consider optimizing the fingerprint database. The positioning accuracy of different algorithms also varies [21].

**Figure 2:**
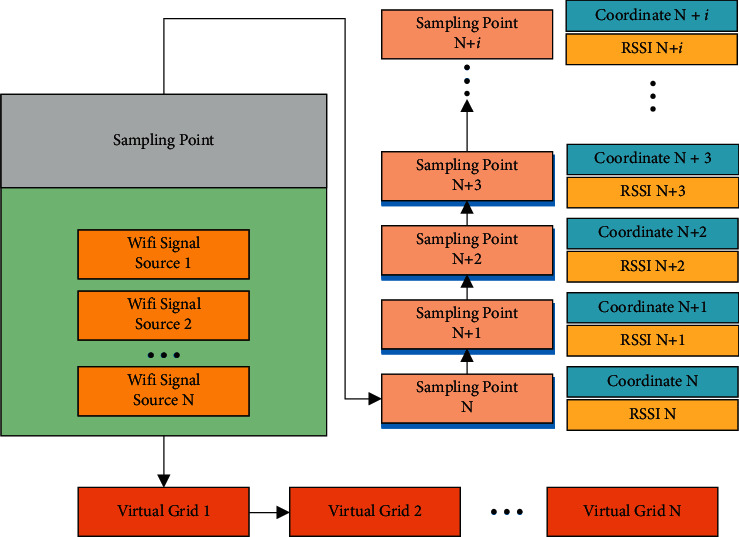
Principles of Wi-Fi-based location fingerprint locating algorithm (i represents the amount of collection points).

The commonly used fingerprint matching and locating algorithms involve the KNN and Nearest Neighbors (NN) algorithms. Still, these two algorithms have the problems of low location accuracy and poor stability. Aiming at the existing issues in the Wi-Fi fingerprint locating algorithm at the present stage, this paper modifies the Wi-Fi signal by mean filtering technology and optimizes the fingerprint database using K-means clustering analysis technology, and the Weight K-Nearest Neighborhood (WKNN) algorithm solves the problems of low positioning accuracy and significant positioning error of the KNN algorithm [22].  Figure 2 illustrates the location fingerprint positioning principle based on Wi-Fi. In Figure 2, the locating algorithm estimates the location of the point to be measured by judging the Wi-Fi signal strength of the power to be measured and comparing the data of the sampling point with the data entered into the fingerprint data set.

**Figure 3:**
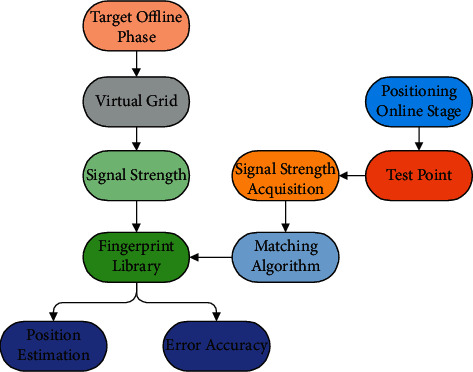
Wi-Fi-based location fingerprint collection process.

The Wi-Fi fingerprint locating process is divided into two stages, offline and online. At the offline stage, the test area is divided into multiple virtual grids; the vertices of the grids are used as signal sampling points to collect the location fingerprint signals of each sampling point; then, the signals are saved into the location fingerprint database. At this stage, the real-time Wi-Fi signal strength is collected from the equipment entering the monitoring area; next, the fingerprint matching algorithm is used to compare the existing fingerprint information in the fingerprint library, and the space coordinates of the test point are calculated [23].  Figure 3 demonstrates the process of location fingerprint collection based on Wi-Fi.

The location fingerprint library collects the data of *n* sampling points in the selected area. The RSSI value of *s* can be obtained at each sampling point as the location fingerprint of the point. By traversing all the sampling points in the area, the *n* location fingerprints in a specific space can be attained and stored in the fingerprint library *FP*. The equation is

(1) $$\begin{array}{c} FP=\left[\begin{array}{cccc} Wifi_{1}^{1} & Wifi_{1}^{2} & \cdots & Wifi_{1}^{s}\\ Wifi_{2}^{1} & Wifi_{2}^{2} & \cdots & Wifi_{2}^{s}\\ \vdots & \vdots & \ddots & \vdots \\ Wifi_{n}^{1} & Wifi_{n}^{2} & \cdots & Wifi_{n}^{s} \end{array}\right]. \end{array}$$

In (1), *Wifi*_*i*_^*s*^ represents the signal strength of the signal source *s* collected at the *i*_*th*_ sampling point [24].

At the offline stage, the location fingerprint information needs to be extracted; combined with the existing information, the location fingerprint information *FP*_*i*_ of the test node can be calculated. Each fingerprint corresponds to a unique location (*x*, *y*). The equation of *FP*_*i*_ is

(2) $$\begin{array}{c} FP_{i}=\left(x_{i},y_{i},Wifi_{i}^{1},Wifi_{i}^{2},\ldots ,Wifi_{i}^{s}\right). \end{array}$$

In (2), (*x*_*i*_, *y*_*i*_) represents the coordinates of the *ith* sampling point in the virtual grid, which can get the location fingerprint $\left(\hat{x},\hat{y}\right)$ at *FP*_*i*_ sampling point *i*. Besides, *k* refers to the number of reference points, which can be written as

(3) $$\begin{array}{c} \left(\hat{x},\hat{y}\right)=\frac{1}{k}\sum_{i}^{k}\left(x_{i},y_{i}\right). \end{array}$$

KNN algorithm can calculate the position coordinates of the sampling point (*x*_*i*_, *y*_*i*_). The average value obtained after multiple position matching is the final estimated position $\left(\hat{x},\hat{y}\right)$ of the test point.

In the actual process of location fingerprint collection, the Wi-Fi signal will be affected by spatial uncertainties such as obstacles and diffraction, causing data fluctuations in the RSSI value and eventually affecting the accuracy of location fingerprint collection. The mean filtering technology is adopted to smooth and filter the instantaneous fluctuations of the collected library data. While processing the data, *k* reference points with the actual distance from the reference point *d* < *δ* are selected. The equation for the reference point is

(4) $$\begin{array}{c} P_{j}\left(x_{j},y_{j}\right)=\sqrt{{\left(x_{i}-y_{j}\right)}^{2}+{\left(x_{i}-y_{j}\right)}^{2}}\leq \delta ,\;\left(j=1,2,\ldots ,k\right). \end{array}$$

In (4), *δ* represents the constant of the distance, which can control the smoothness of the mean smoothing filter and is generally set to a value slightly larger than the average distance of the reference point [25].

The RSSI values of the Wi-Fi signals of the *k* nearest reference points are averaged, which is the RSSI value of point *P*_*i*_(*x*_*j*_, *y*_*j*_), expressed as

(5) $$\begin{array}{c} \text{RSSI}{\left(P\right)}_{i}=\sum_{j=1}^{j=k}\text{RSSI}\left(P_{j}\right). \end{array}$$

**Figure 4:**
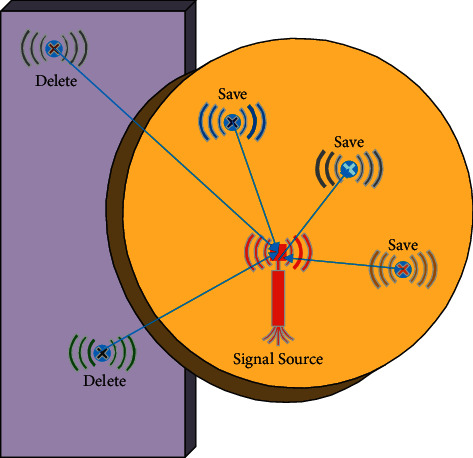
Principles of K-means clustering algorithm for noise reduction.

Location fingerprint data are classified by a clustering algorithm to optimize the fingerprint database. The signal values of Wi-Fi signal data collected at each sampling point are clustered. Assuming that there are *m* sampling points and *n* Wi-Fi signal sources, there are *m* × *n* location fingerprint clusters.  Figure 4 shows the principle of the K-means clustering algorithm for noise reduction of location fingerprints. The sampling point is considered a noise point when the RSSI value of the sampling point minus the center value Ψ of clustering is greater than the signal noise threshold. The point is deleted from the location fingerprint database. The optimized location fingerprint database is obtained after traversing the location fingerprints.  Figure 4 shows the principles of K-means clustering noise reduction. The K-means clustering algorithm for noise reduction can divide the collected data and delete some noise points with large deviations to obtain more effective and reasonable data.

In the actual locating stage, KNN matches the real-time collected Wi-Fi signal strength with the signal in the fingerprint library, finds the most similar sampling point of the fingerprint data, and uses the actual position of the sampling point as the estimated position of the test point to estimate the Wi-Fi fingerprint position [26]. KNN sorts the signal strength of the test point and the Euclidean distance of the signal strength in the location fingerprint database during calculation. The coordinates of the first *K* (*K* ≥ 2) sampling points with the smallest Euclidean distance are selected. The coordinates of the K neighbouring sampling points are averaged, which are the final coordinate position of the test point, as

(6) $$\begin{array}{c} \left(x,y\right)=\frac{1}{K}\sum_{j=1}^{K}\left(x_{j},y_{j}\right). \end{array}$$

However, the reference fingerprint data of KNN is single, leading to large errors in the locating results. KNN is optimized by weighting to understand its locating error, namely, the WKNN algorithm. In the present work, the weight coefficient *δ* is introduced and applied to different references in the location fingerprint library to distinguish the importance of fingerprints in during the matching process. WKNN uses the contribution of each sampling point to determine the location of the test point as a weighting factor. The smaller the Euclidean distance between the sampling point and the test point, the greater the weight value of the location fingerprint of the sampling point. The equation is

(7) $$\begin{array}{c} \left(x,y\right)=\sum_{j=1}^{K}\frac{1/dis_{j}+\varepsilon }{\sum_{j=1}^{K}dis_{j}+\varepsilon }\left(x_{j},y_{j}\right). \end{array}$$

In (7), *dis*_*j*_ represents the Euclidean distance between the sampling point and the test point, *K* indicates the number of selected sampling points with the closest Euclidean distance, and *ε* refers to an integer with a small but nonzero number. To prevent the denominator from being zero, the strength of the received wireless signal will decrease as the Euclidean distance increases. Hence, the weight coefficient is inversely proportional to the Euclidean distance of the sampling point [27]. The equation to calculate the location fingerprint data of the sampling point is

(8) $$\begin{array}{c} \delta_{i}=\frac{\sigma_{i}}{\sum_{i=1}^{k}R_{i}}. \end{array}$$

(9) $$\begin{array}{c} \sigma_{i}=\sqrt{\frac{\sum_{j=1}^{n}{\left(RSSI_{ij}-{\bar{RSSI}}_{i}\right)}^{2}}{n}}. \end{array}$$

(10) $$\begin{array}{c} R_{i}=\frac{\sigma_{i}}{{\bar{RSSI}}_{i}}. \end{array}$$

In (8)–(10), *σ*_*i*_ represents the standard deviation of the RSSI value in cluster *i*, $\bar{RSSI_{i}}$ denotes the mean value of RSSI in the *i*_*th*_ cluster, the larger the *σ*_*i*_, the greater the difference between the RSSI value in the cluster and the mean value; *n* represents the coefficient of dispersion and *R*_*i*_ represents the degree of dispersion between the standard deviation and the mean value of the RSSI value of cluster *i*.

## 4. Building Energy Consumption Optimization Based on NSGA-II

The utilization rate of building energy consumption has been increasing as China's modernization process gets accelerated. Due to the complexity of building design, traditional design methods cannot meet the multiple target needs of modern building design; the qualitative and quantitative methods cannot optimize the design and research of building physical problems such as building energy consumption [28]. The theoretical basis to calculate the building energy consumption is the first law of thermodynamics. During calculation, the thermal balance equation is established to obtain the thermal parameters and air condition parameters of each room in the building. The heat change of the indoor air heat exchange and air conditioning are calculated, as well as the cooling capacity that the system needs to provide. If the temperature in the building room is not determined, the heat exchange law of the air conditioner and the air temperature inside the building need to be calculated. Without considering the heat storage capacity of the indoor air, it is also necessary to calculate the temperature change of the air through the indoor energy balance state [29].

From a cybernetics point of view, the building energy system is a multivariable nonlinear complex system. The building energy-saving goals also need to be implemented in terms of optimization control of the building environment and prediction and management of building energy. The space of modern buildings becomes increasingly larger, the internal structure becomes increasingly complex, and the concentration of equipment keeps increasing, which makes it hard to accurately calculate the building energy consumption. The traditional calculation method uses the air conditioning load to calculate the indoor steady-state heat transfer process. The heat that maintains the building's potential energy temperature and humidity state is calculated by simplifying the external conditions. It can be divided into calculating long-term load characteristics and calculating instantaneous maximum load and design load [30, 31]. However, the thermal characteristics of the building structure can affect the analysis results of the building energy consumption. Hence, it is necessary to calculate the energy consumption of the building's potential energy air conditioning system more accurately.

**Figure 5:**
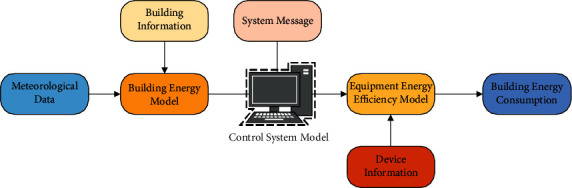
Analysis basis of dynamic building energy consumption.

With the improvement of energy consumption calculation accuracy, traditional calculation methods cannot meet the research needs of building interior design. The development of computer technology and multiobjective calculation optimization technology provides effective support for the research of dynamic building energy consumption simulation. The estimation technology accurately calculates the hourly building energy consumption required by the building's indoor environment by analyzing factors such as building indoor air conditions, indoor personnel activities, and outdoor weather data. Its core is the dynamic calculation process of energy consumption, making the simulation process of building energy consumption more accurate [32]. The dynamic energy consumption simulation process in the building's indoor environment includes the building energy consumption model, air conditioning system model, and equipment energy efficiency model.  Figure 5 describes the logical relationships among the three.

During building design and energy consumption analysis, many load-related problems can occur and need to be solved under specific conditions. Since the building organization is a complex organic whole, the optimization of one aspect of the building is likely to lead to imbalances in other aspects. Hence, in the mutual influence target award, simply pursuing the optimization of a target can lead to the imbalance of the overall building state. Consequently, it is necessary to introduce a multiobjective optimization design to meet the multiobjective coordination optimization in the building at the same time [33]. Regarding the problems and contradictions in multiobjective optimization, it is necessary to convert multiple subobjectives into corresponding functional problems and combine experience and actual needs to choose the optimal solution. Although the actual needs can be met, the memory obtained in this way is far from the actual optimization results.

**Figure 6:**
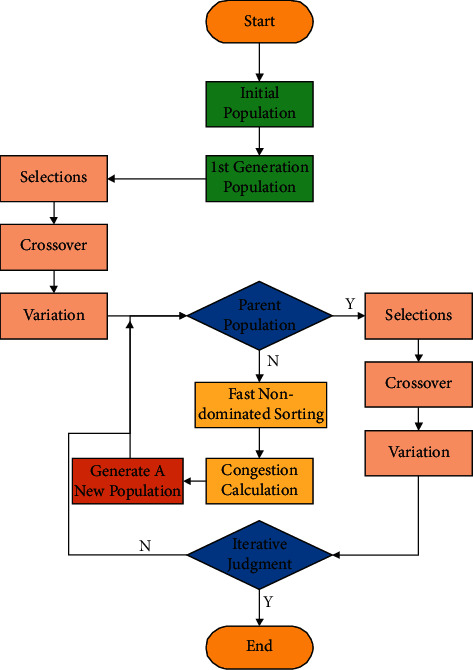
Algorithm flow of NSGA-II.

NSGA-II randomly generates a population of size *N*; after nondominated sorting, the genetic algorithm is adopted to obtain the first generation population through crossover, mutation, and selection. Since the second-generation population, the parent population and the offspring population are combined for fast nondominated sorting; then, the crowding degree of the population individuals in each nondominated layer is calculated. According to the nondominated relationship and the crowding degree of the population individuals, suitable population individuals are selected to form a new parent population [34]. Afterward, a new offspring population is generated through crossover, mutation, and selection. This process is repeated until the experimental results obtained can meet the set termination conditions.  Figure 6 shows the flowchart of NSGA-II.

A solution set is usually obtained during multiobjective optimization. Each section in the solution set is a solution of multiobjective optimization. Therefore, while optimizing the objective function of a solution, it will inevitably cause the degradation of other objective functions. This solution set is called the Pareto optimal solution set. An effective approach for objective optimization is to study a set of solutions that represent the Pareto optimal set. Therefore, objectives that should be obtained through multiobjective optimization include the following: (1) the representative Pareto frontier should be as close as possible to the true Pareto frontier; the rector front, ideally, should be a subset of the Pareto optimal set. (2) The solution to the representative Pareto set should be evenly distributed and dispersed on the Pareto front to provide decision-makers with a realistic picture of the trade-offs.

## 5. Algorithm Testing and Simulation

### 5.1. Effects of Mean Filtering and Clustering Optimization

**Figure 7:**
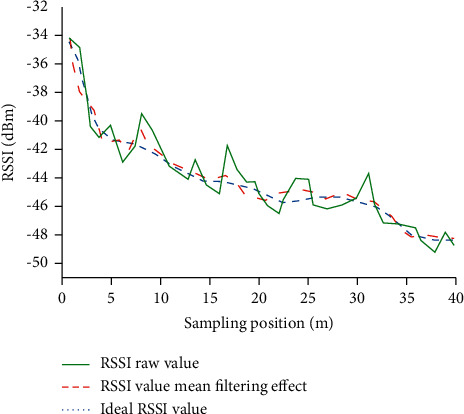
Comparison of filtering algorithm effects.

Regarding the Wi-Fi signal fluctuations in the building, mean filtering is used to process the collected location fingerprint signal strength to improve the accuracy of the algorithm locating.  Figure 7 shows the RSSI effect after mean filtering.

Figure 7 suggests that the RSSI value continues to weaken with the increase in the locating distance. Due to the influence of the building space characteristics such as diffraction and obstacles, the original RSSI strength value will produce larger numerical fluctuations. Changes in the theoretical RSSI strength value are pretty smooth. Although RSSI strength value can be affected by the characteristics of the indoor space, the overall value fluctuates little. Compared with the theoretical value, the RSSI strength after mean filtering fluctuates; nevertheless, the optimized effect is significantly better than the collected original value of RSSI strength. Hence, the RSSI value optimized by mean filtering can better solve Wi-Fi signal fluctuation in the building.

**Figure 8:**
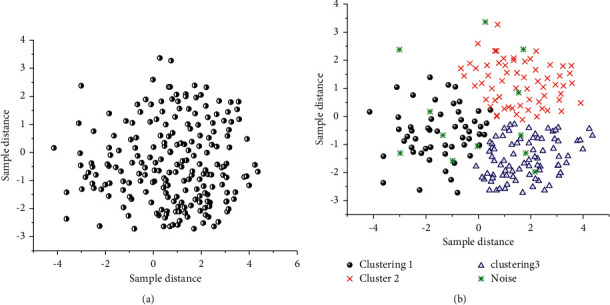
Classification effect of K-means clustering algorithm on fingerprint database. (a) Sample library before cluster optimization. (b) Sample library after cluster optimization.

To verify the optimization effect of the location fingerprint library based on the K-means cluster analysis, the locating scenario in the test area under study uses the same locating algorithm to collect location fingerprint data and aggregate it into a fingerprint library. The K-means clustering algorithm is employed to optimize the fingerprint database. The experimental records are compared, and the results of fingerprint database clustering are displayed in Figure 8.

As shown in Figure 8, samples of the original fingerprint library are scattered, with some data noises. After the data are optimized by the K-Means clustering algorithm, the discrete fingerprint samples can be well classified according to the data features. The data features of the location fingerprint library can be effectively described in this way.

**Figure 9:**
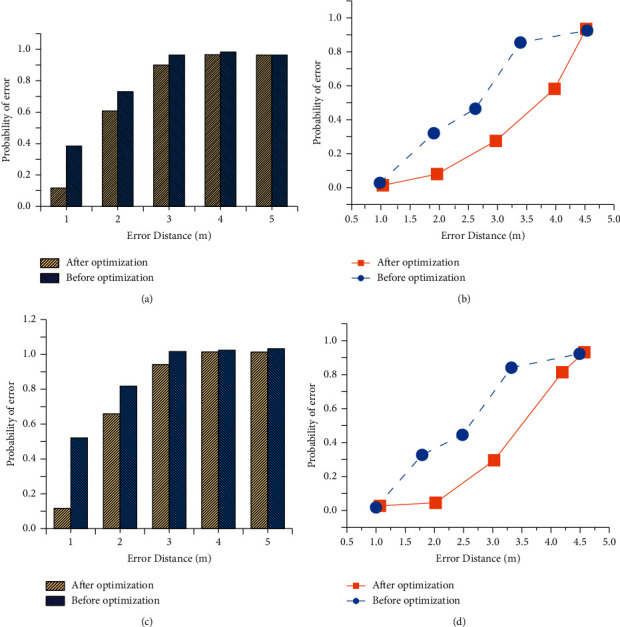
Comparison of the locating algorithm effect after fingerprint optimization. (a) Test of the first group, 20 nodes. (b) Test of the second group, 20 nodes. (c) Test of the first group, 50 nodes. (d) Test of the second group, 50 nodes.

This experiment randomly selects and compares the data to verify the optimization effect of the K-means clustering algorithm on the fingerprint database.  Figure 9 shows the comparison of the effects before and after fingerprint database optimization.

According to Figure 9, with the growth of error distance, the recognition error rate of the algorithm is also increasing. The recognition error rate of the optimized algorithm decreases and the positioning accuracy of target recognition is improved. The error rate within 2 m is about 60%. Moreover, the nodes 20 and 50 have little impact on the recognition effect of the positioning algorithm, so the number of sampling points in the space will not have much impact on the calculation results of the actual positioning algorithm. The fingerprint database optimized by the K-means clustering algorithm can significantly improve the positioning effect of the positioning algorithm. Besides, the error probability increases gradually with the increase in positioning distance. Therefore, when locating the test point, it is necessary to select the nearest virtual grid for sample signal acquisition to reduce the positioning error of the locating algorithm.

### 5.2. Experimental Analysis of Locating Algorithm

**Figure 10:**
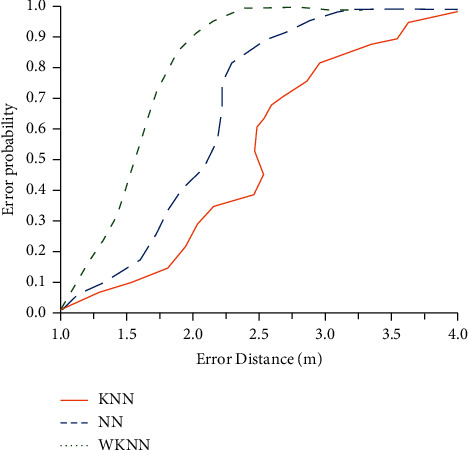
Accuracy comparison of locating algorithms.

The established experimental scenario is used to compare the WKNN algorithm, KNN algorithm, and NN algorithm to validate the positioning accuracy of the WKNN algorithm for indoor test points.  Figure 10 illustrates the experimental results.

As can be seen from Figure 10, the convergence distance of the WKNN algorithm is 2 m; the convergence distances of the KNN algorithm and NN algorithm are 3 m and 3.8 m, respectively. Therefore, the KNN algorithm has better positioning accuracy and better positioning effect compared with similar algorithms. From the error curve, the positioning accuracy of KNN algorithm and NN algorithm fluctuates greatly and cannot get stable positioning results. In contrast, the WKNN algorithm achieves a smoother error accuracy curve, which has better positioning stability than KNN algorithm and NN algorithm. Therefore, the WKNN algorithm is superior to similar algorithms in terms of positioning accuracy and positioning stability.

## 6. Conclusions

The impact of people's living behavior on building design and energy consumption is discussed to study the problems of building interior design and energy consumption optimization under the background of modernization. Aiming at the mobility of people in building space, a location fingerprint locating algorithm based on Wi-Fi is proposed to identify the location of people in the building and judge the mobility of people in the building. Besides, a Nondominated Sorting Genetic algorithm is proposed to optimize the energy consumption in the building to reduce building internal energy consumption to attain the optimal solution set of the building indoor models. Finally, the algorithm designed here is simulated and tested. The experimental results indicate that the location fingerprint of the sampling point optimized by the mean filtering algorithm and K-means clustering analysis has been significantly improved. This WKNN positioning method effectively reduces the data disturbance caused by the indoor environment and improves the positioning accuracy of the locating algorithm. The error rate within 2 m is about 60%. Compared with other algorithms, the WKNN locating algorithm has better location stability, and the accuracy of indoor location problems can effectively identify and analyze the indoor environment. The research reported here proves the effectiveness of indoor positioning algorithms to building energy consumption regulation and optimizes the designed algorithm, providing a theoretical and application basis for related research. However, there are some deficiencies in this paper. The indoor locating algorithm has high computational complexity and delay in the positioning process, affecting the accuracy of the positioning algorithm. Therefore, the follow-up research will transfer the computing service to the server to improve the computing performance of the locating algorithm. Moreover, the performance of the objective optimization model will be improved to obtain more effective energy consumption optimization results to collect indoor personnel flow information.

## Data Availability

The data used to support the findings of this study are included within the article.
